# Segmental snare traction technique: A stepwise snaring strategy for helix-fixed leadless pacemaker implantation

**DOI:** 10.1016/j.hrcr.2025.10.008

**Published:** 2025-10-14

**Authors:** Yoshikazu Suzuki, Yuya Nakamura, Haruka Miyazaki, Shuhei Arai, Taku Asano, Toshiro Shinke

**Affiliations:** Division of Cardiology, Department of Medicine, Showa University School of Medicine, Tokyo, Japan

**Keywords:** Leadless pacemaker, Dual-chamber pacing, Helix-fixed system, Snare technique, Tricuspid valve

**Key Teaching Points**

• Sequential snare traction at distal, midshaft, and proximal ventricular leadless pacemaker segments enables controlled navigation through the tricuspid valve in anatomically constrained patients.
• Each traction point offers distinct biomechanical advantages: distal for valve crossing, midshaft for curvature adjustment and enhanced forward advancement, and proximal for septal alignment.
• The segmental snare traction technique can serve as a practical bailout strategy in cases where conventional delivery fails owing to right heart anatomic challenges.

## Introduction

Leadless pacemakers have emerged as an alternative to transvenous systems, reducing lead- and pocket-related complications.^1^ The development of dual-chamber leadless systems, such as the Aveir DR (Abbott, Symar, CA), has further expanded indications to include patients with atrioventricular (AV) block who require AV-synchronous pacing.^2^ However, implantation of the ventricular leadless pacemaker (vLP) may be challenging in patients with small right atrial (RA) chambers, a condition more frequently encountered in those without atrial fibrillation,^3^ owing to the difficulty in advancing the delivery catheter (DC) through the tricuspid valve (TV).

Previous reports have described the use of snare-assisted techniques to facilitate the implantation of helix-fixed leadless pacemakers in anatomically challenging cases. In some of these cases, the snare position and traction force were adjusted in response to the procedural course, resulting in a functionally stepwise approach.^4^^,^^5^ In this report, we describe 2 cases in which a stepwise snaring approach—using traction at 3 distinct positions (distal, midshaft, and proximal) on the vLP—enabled controlled passage through the TV and successful implantation in the right ventricular (RV) septum.

## Case presentation

### Segmental snare traction technique

The segmental snare traction technique (SSTT) is a method developed to facilitate controlled advancement and deployment of a vLP through the TV in patients with anatomically constrained right heart structures. The technique comprises the following access and preparation steps:

1. Access and setup: The procedure is performed by 2 electrophysiologists using a biplane fluoroscopy system under local anesthesia with light, titrated conscious sedation using midazolam. Continuous hemodynamic and respiratory monitoring is maintained, and right femoral venous access is obtained.

**Figure 1:**
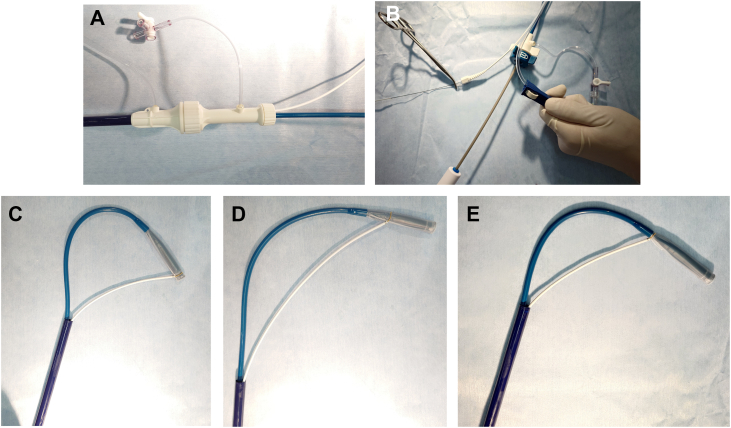
Demonstration of the segmental snare traction technique. **A:** Snare introduced alongside the delivery catheter via the valve bypass tool. **B:** Valve bypass tool with clamp fully opened to minimize backflow. **C:** Distal segment grasped to facilitate passage through the tricuspid valve. **D:** Midshaft grasped to increase the curvature radius and improve forward force. **E:** Proximal segment grasped to redirect the ventricular leadless pacemaker toward the right ventricular septum.

A 27F introducer sheath is inserted, and a 90-degree single-loop snare is advanced alongside the Aveir vLP DC via a valve bypass tool (Figure 1A). To minimize retrograde blood flow, the clamp on the valve bypass tool is fully opened (Figure 1B).

2. Segmental traction strategy: This technique involves sequential snaring of 3 distinct portions of the vLP body:
  • Distal segment: the distal tip of the vLP is grasped with the snare to facilitate passage through the TV when standard advancement proves difficult (Figure 1C). The DC forms an S-shaped curvature, with its bend positioned near the right hepatic vein. Traction is applied to the distal segment while advancing the DC, allowing the system to cross the superior margin of the tricuspid annulus with a smaller radius of curvature.
  • Midshaft segment: Grasping the midshaft portion of the vLP increases the radius of curvature at the nondeflectable segment of the DC and enhances forward transmission of force (Figure 1D). This facilitates advancement toward the RV apex.
  • Proximal segment: Grasping the proximal portion of the vLP—occasionally including the docking cap—allows redirection of the vLP toward the RV septum (Figure 1E). This maneuver causes the DC to bend counterclockwise within the RA, enabling the vLP to shift from a lateral RV orientation toward a septal trajectory.

In SSTT, curvature is shaped primarily by snare traction, and the deflection lever is used sparingly. This single-curvature policy reduces complexity and enables sharper, more precise angulation. The protective sleeve position and docking integrity are intermittently confirmed under fluoroscopy to mitigate inadvertent deployment or docking failure.

### Case 1

An 89-year-old woman with a history of osteoporosis presented to our outpatient clinic with exertional dyspnea. Electrocardiography revealed advanced AV block with a heart rate of 36 beats/min. Her height was 146 cm, weight 52 kg, body mass index 24.4 kg/m^2^, and body surface area 1.45 m^2^, with kyphosis. Transthoracic echocardiography showed a preserved left ventricular ejection fraction of 59% without significant valvular disease. On hospital day 2, following the patient’s preference, a decision was made to implant an Aveir DR leadless pacemaker to treat the advanced AV block.

Under local anesthesia, a 27F introducer sheath was inserted via the right femoral vein. Right heart angiography revealed that the inferior vena cava (IVC) connected to the RA from a posterior direction with marked tortuosity. As a result, the vLP exited the delivery sheath with a posterior orientation toward the lateral wall of the RA, making passage across the TV difficult. Despite 20 minutes of manipulation, the system could not be advanced into the RV.

**Figure 2:**
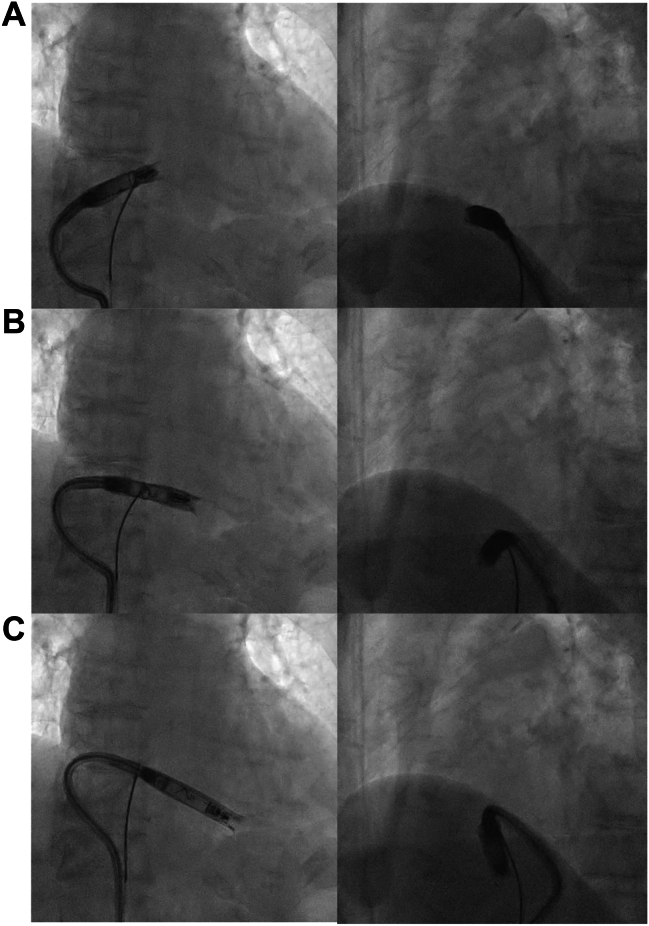
Fluoroscopic images from the first case demonstrating the 3 traction positions of the segmental snare traction technique. Each panel includes anteroposterior (AP) and left anterior oblique (LAO) 45° views. **A:** Distal snare traction formed a tight curvature in the delivery catheter, facilitating tricuspid valve (TV) crossing. **B:** Midshaft snare traction increased the radius of curvature at the nondeflectable portion of the catheter and improved forward advancement toward the right ventricle (RV). **C:** Proximal snare traction near the docking cap induced counterclockwise rotation of the delivery catheter, redirecting the ventricular leadless pacemaker (vLP) from the right ventricular free wall toward the septum. AP = anteroposterior; LAO = left anterior oblique; RV = right ventricle; TV = tricuspid valve; vLP = ventricular leadless pacemaker.

A 90-degree single-loop snare was introduced to assist in the delivery. The snare was positioned at the IVC–RA junction and was used to capture the vLP as it exited the delivery sheath into the RA. The midshaft portion of the vLP was grasped, and traction was applied. However, owing to the large radius of curvature at the nonarticulating segment of the vLP and DC, the system became lodged against the superior margin of the tricuspid annulus and could not cross the valve. Subsequently, the distal portion of the vLP was grasped, and forward pressure was applied to the DC, which reduced the radius of curvature and enabled the vLP to cross the TV (Figure 2A). However, the system remained oriented superiorly toward the superior vena cava, resulting in inadequate forward force toward the RV apex. To address this, the midshaft was grasped to increase the curvature radius of the nondeflectable segment and improve transmission of forward force (Figure 2B). Because the IVC enters the RA from a posterior direction, the delivery trajectory caused the vLP to face the anterior wall of the RV. Therefore, the proximal portion of the vLP was finally grasped, including the docking cap. This maneuver rotated the DC along the lateral wall of the RA, redirecting the vLP toward the RV septum (Figure 2C). Once an orientation toward the septal aspect of the inferior RV wall was confirmed, electrical premapping was performed and the vLP was successfully fixated. Immediately after implantation, the ventricular pacing threshold was 0.5 V at 0.4 ms pulse width, R-wave amplitude was 6.8 mV, and impedance was 340 Ω. After the introduction of the SSTT, vLP implantation was completed within 13 minutes. The fluoroscopy time during snare-assisted manipulation was 4 minutes, with a total dose–area product of 9 Gy·cm^2^. Subsequently, the atrial leadless pacemaker was implanted without complications.

### Case 2

A 75-year-old woman with a medical history of cervical cancer and hypertension was emergently transported to our hospital after experiencing a syncopal episode. The initial electrocardiogram demonstrated complete AV block with a ventricular rate of 40 beats/min. Her height was 150 cm, weight 42 kg, body mass index 18.7 kg/m^2^, and body surface area 1.34 m^2^. Transthoracic echocardiography revealed a preserved left ventricular ejection fraction of 62%, without significant valvular disease. On the day of admission, a temporary pacemaker was implanted via the right internal jugular vein. Coronary angiography was also performed, which showed no significant coronary artery stenosis. Given the patient’s preference for a leadless pacemaker, implantation of the Aveir DR system was scheduled on hospital day 4 for treatment of complete AV block.

Under local anesthesia, the 27F introducer sheath was introduced via the right femoral vein. Initial right heart angiography from the IVC to the RV revealed an IVC connecting to the RA from a posterior direction, with a dilated aorta displacing the TV inferiorly. Passage through the TV with standard DC manipulation was unsuccessful; therefore, after 25 minutes of unsuccessful attempts, vLP implantation was performed using the SSTT via a 90-degree single-loop snare.

**Figure 3:**
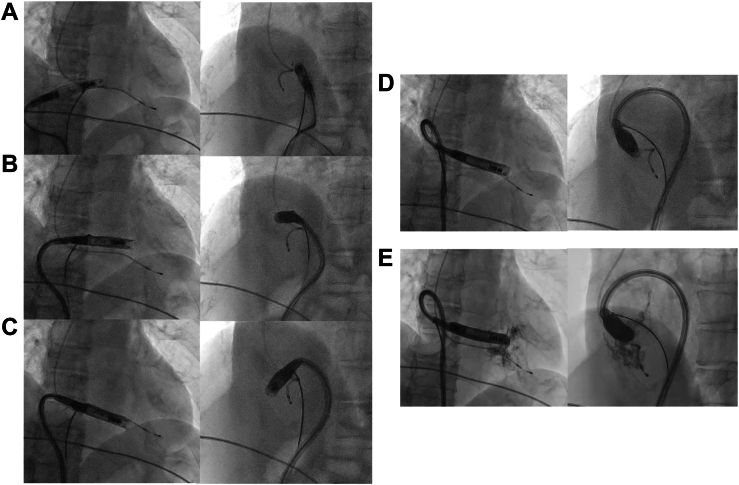
Fluoroscopic images from the second case showing progressive changes in catheter orientation during sequential snare traction. **A:** Distal snare traction formed a tight curvature in the delivery catheter, aiding tricuspid valve (TV) crossing. **B:** Midshaft snare traction increased the radius of curvature at the nondeflectable portion of the catheter and improved forward pushability toward the right ventricle (RV). **C:** Proximal snare traction near the docking cap induced counterclockwise rotation of the catheter along the posterior, lateral, and anterior walls of the right atrium (RA). **D:** More proximal snare traction further redirected the tip of the ventricular leadless pacemaker (vLP) toward the right ventricular septum. **E:** Contrast injection from the protective sleeve at the same position as in panel D confirmed that the vLP tip was oriented toward the septum. RA = right atrium; RV = right ventricle; TV = tricuspid valve; vLP = ventricular leadless pacemaker.

First, the distal segment of the vLP was grasped, and the DC was shaped into an S-shaped curve, with the primary bend positioned at the right hepatic vein, enabling the tip to pass over the superior edge of the TV without excessive stress (Figure 3A). The snare was then repositioned to the midshaft segment, increasing the radius of curvature at the nondeflectable portion of the DC and thereby improving forward force transmission toward the RV apex (Figure 3B). In this phase, the S-shaped curve was maintained within the RA. Because the posterior IVC–RV connection caused the vLP to orient toward the RV free wall, the snare was advanced proximally to grasp the docking cap region (Figure 3C). Gentle traction at this point induced a counterclockwise rotation of the DC along the posterior, lateral, and anterior walls of RA, successfully redirecting the vLP toward the RV septum (Figure 3D). After loosening the snare, contrast injection through the protective sleeve confirmed that the vLP was oriented toward the RV septum and appropriately positioned (Figure 3E). With the S-shaped curve now directed toward the septal aspect of the inferior RV wall (this sequence of catheter manipulation and redirection, as illustrated in Figure 3, is demonstrated in real time in Supplemental Video 1), electrical premapping was performed, followed by fixation of the vLP into the myocardium. Immediately after implantation, the ventricular pacing threshold was 0.5 V at 0.4 ms pulse width, R-wave amplitude was 9.6 mV, and impedance was 750 Ω. From the start of the SSTT application, vLP implantation was completed within 15 minutes. The fluoroscopy time during snare-assisted manipulation was 5 minutes, with a total dose–area product of 11 Gy·cm^2^.

Subsequently, an atrial leadless pacemaker was implanted without complications, and the procedure was completed uneventfully.

## Discussion

Implantation of leadless pacemakers can be challenging in patients with small RA and RV chambers or a posteriorly oriented IVC. In such cases, manipulation of the vLP within the RA is restricted, and the DC may face the free wall of the RV, making it difficult to cross the tricuspid annulus or to achieve fixation toward the RV septum.

To overcome these anatomic limitations, snare-assisted techniques have been reported. Although most reported techniques involve single-point traction, such approaches provided limited control in the 2 cases described here.

**Figure 4:**
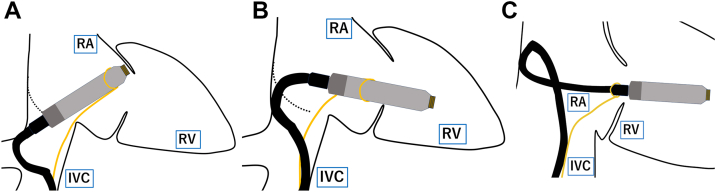
Schematic illustration of the segmental snare traction technique showing 3 sequential traction sites. **A:** Distal snare traction results in a small radius of curvature in the delivery catheter, facilitating tricuspid valve (TV) crossing, with the S-shaped bend positioned at the level of the right hepatic vein. **B:** Midshaft snare traction increases the radius of curvature at the nondeflectable portion of the catheter. In addition, it improves forward transmission of force toward the right ventricular (RV) apex. **C:** Proximal snare traction near the docking cap allows counterclockwise rotation of the delivery catheter along the lateral and anterior walls of the right atrium (RA), redirecting the ventricular leadless pacemaker (vLP) from the right ventricular free wall toward the septum. IVC = inferior vena cava; RA = right atrium; RV = right ventricle; TV = tricuspid valve; vLP = ventricular leadless pacemaker.

The SSTT offers a structured, stepwise strategy in which traction is applied sequentially at 3 different segments of the vLP—distal, midshaft, and proximal. This enables dynamic adjustment of the delivery system to navigate the TV and align the vLP toward the septum. Distal snaring facilitates passage through the TV with a tight curvature (Figure 4A), midshaft traction improves forward force transmission (Figure 4B), and proximal traction allows redirection from the RV free wall to the septum via counterclockwise rotation of the DC within the RA (Figure 4C).

With the emergence of dual-chamber leadless systems, implantation procedures are likely to be performed in a broader patient population, including those with smaller or anatomically constrained right heart structures. In such scenarios, delivery of the vLP may become increasingly difficult. The SSTT may serve as a practical and reproducible bailout strategy to facilitate controlled navigation and positioning of the vLP in these anatomically challenging cases.

### Limitations and clinical implications

The SSTT requires 2 operators who clearly understand the procedural steps and coordinate closely throughout the implantation. This technique is intended as a bailout strategy rather than a standard approach and should be considered only in cases where conventional delivery of the vLP proves difficult owing to anatomic constraints. Although no premature release, docking malfunction, or failure of the standard release steps occurred in the present cases, snare traction could theoretically compromise docking integrity or, conversely, hinder the usual undocking/release sequence. Operators should monitor the protective sleeve and docking under fluoroscopy. In addition, this report is limited to 2 cases; further clinical experience is necessary to evaluate the reproducibility, safety, and generalizability of this method.

## Conclusion

The SSTT provides a structured and reproducible method for navigating challenging right heart anatomies during dual-chamber leadless pacemaker implantation. By sequentially applying traction at the distal, midshaft, and proximal segments of the vLP, this technique facilitates controlled passage through the TV and optimal positioning toward the RV septum. In anatomically constrained patients where conventional delivery fails, this approach may serve as an effective bailout strategy to enable successful device implantation.

## Disclosures

The authors have no conflicts of interest to disclose.

## References

[1] Knops R.E., Reddy V.Y., Ip J.E. (2023). A dual-chamber leadless pacemaker. N Engl J Med.

[2] Neuzil P., Hubbard C., Doshi R.N. (2024). Implantation techniques for a helix-fixation dual-chamber leadless pacemaker. Heart Rhythm.

[3] Gunturiz-Beltrán C., Nuñez-Garcia M., Althoff T.F. (2022). Progressive and simultaneous right and left atrial remodeling uncovered by a comprehensive magnetic resonance assessment in atrial fibrillation. J Am Heart Assoc.

[4] Shichijo K., Oka T., Yoshida A., Sekihara T., Sakata Y. (2025). Helix-fixed leadless pacemaker implantation requiring the gooseneck snaring technique for passage through the tricuspid valve: a case series. HeartRhythm Case Rep.

[5] Shah H., Winner M., Patel D. (2025). Snare-assisted flexion of 27-French leadless pacemaker delivery sheath: a case report. J Cardiovasc Electrophysiol.

