# Supplementary material for: Segmental snare traction technique: A stepwise snaring strategy for helix-fixed leadless pacemaker implantation
Source: HeartRhythm Case Rep. 2025 Oct 14;11(12):1383–7. doi: 10.1016/j.hrcr.2025.10.008 (PMC12805288; doi:10.1016/j.hrcr.2025.10.008)
Supplement: Supplementary Video 1 [file mmc2.docx]

**Supplementary Video 1. Real-time fluoroscopic demonstration of the Segmental Snare Traction Technique in Case 2.**

This video shows the sequential snaring of the distal, midshaft, and proximal segments of the ventricular leadless pacemaker (vLP), followed by contrast injection confirming its final orientation toward the right ventricular septum.
